# Identification of an ADAM17 Cleavage Region in Human CD16 (FcγRIII) and the Engineering of a Non-Cleavable Version of the Receptor in NK Cells

**DOI:** 10.1371/journal.pone.0121788

**Published:** 2015-03-27

**Authors:** Yawu Jing, Zhenya Ni, Jianming Wu, LeeAnn Higgins, Todd W. Markowski, Dan S. Kaufman, Bruce Walcheck

**Affiliations:** 1 Department of Veterinary and Biomedical Sciences, University of Minnesota, Minneapolis and St. Paul, Minnesota, United States of America; 2 Department of Medicine, Stem Cell Institute, University of Minnesota, Minneapolis and St. Paul, Minnesota, United States of America; 3 Department of Biochemistry, Molecular Biology and Biophysics, University of Minnesota, Minneapolis and St. Paul, Minnesota, United States of America; University Paris Sud, FRANCE

## Abstract

CD16a and CD16b are IgG Fc receptors expressed by human natural killer (NK) cells and neutrophils, respectively. Both CD16 isoforms undergo a rapid down-regulation in expression by ADAM17-mediated proteolytic cleavage upon cell activation by various stimuli. We examined soluble CD16 released from activated NK cells and neutrophils by mass spectrometric analysis, and identified three separate cleavage sites in close proximity at P1/P1′ positions alanine195/valine196, valine196/serine197, and threonine198/isoleucine199, revealing a membrane proximal cleavage region in CD16. Substitution of the serine at position 197 in the middle of the cleavage region for a proline (S197P) effectively blocked CD16a and CD16b cleavage in cell-based assays. We also show that CD16a/S197P was resistant to cleavage when expressed in the human NK cell line NK92 and primary NK cells derived from genetically-engineered human induced pluripotent stem cells. CD16a is a potent activating receptor and despite blocking CD16a shedding, the S197P mutation did not disrupt IgG binding by the receptor or its activation of NK92 cells by antibody-treated tumor cells. Our findings provide further characterization of CD16 cleavage by ADAM17 and they demonstrate that a non-cleavable version of CD16a can be expressed in engineered NK cells.

## Introduction

The human IgG Fc receptor III (FcγRIII, FCGR3, or CD16) consists of two isoforms (CD16a and CD16b) that are encoded by two highly homologous genes [1]. CD16b is glycosylphosphatidylinositol (GPI)-anchored to the cell membrane and is expressed primarily by neutrophils [2]. CD16b is a low affinity receptor that binds aggregated IgG and is important for immune complex clearance and neutrophil attachment to immobilized immune complexes on endothelial surfaces [3, 4]. In contrast, CD16a is a transmembrane protein and also the only FcγR expressed by NK cells [4]. This receptor binds monomeric IgG attached to target cells to facilitate antibody-dependent cell-mediated cytotoxicity (ADCC), a key effector mechanism of NK cells [5].

Both CD16 isoforms undergo very rapid and efficient proteolytic cleavage upon neutrophil and NK cell activation by various stimuli [6–10]. We have directly demonstrated that ADAM17 (A Disintegrin And Metalloprotease-17) is the primary protease mediating CD16b cleavage [9]. We as well as others have also reported that ADAM17 cleaves CD16a in activated NK cells [9–12], though Membrane-Type 6 Matrix Metalloproteinase may have a role this process as well [13].

CD16 cleavage occurs proximal to the cell membrane, resulting in the release of an intact and functional receptor [6, 7, 9, 14]. Soluble CD16 can be detected in the plasma of healthy individuals [6, 9, 15], which is predominantly derived from neutrophils and composed of CD16b [6]. Moreover, plasma levels of CD16 were significantly reduced in patients treated with an ADAM17 inhibitor [9]. These findings indicate that CD16 cleavage by ADAM17 is a physiological process.

We sought to characterize the site of CD16 proteolysis upon its release from human NK cells and neutrophils. We report for the first time the presence of a short cleavage region in the membrane proximal portion of CD16. Exchanging a serine residue in this region with a proline disrupted the cleavage of CD16a and CD16b in transfected cells. Moreover, the engineered mutation in CD16a prevented its down-regulation in the human NK cell line NK92 and in primary NK cells derived from human induced pluripotent stem cells (iPSCs) when activated with various stimuli. The mutation, however, did not disrupt IgG binding or cell activation by CD16a, indicating that the receptor remained functional.

## Materials and Methods

### Mass spectrometry analysis

Peripheral blood collection from healthy individuals was performed in accordance with protocols approved by the University of Minnesota Institutional Review Board according to protocol # 9708M00134. Participants provided written agreement to donate blood by signing an IRB-approved consent form. Human neutrophil and NK cell isolation was performed as previously described [9, 16, 17]. Enriched neutrophils or NK cells (1x10^7^/ml in PBS; Mediatech, Hevdon, VA) were activated with PMA (15ng/ml or 50ng/ml, respectively; Sigma, St. Louis, MO) for 30 minutes at 37°C. Cell supernatants were filtered (0.45μm pore size) and CD16 was immunoprecipitated using the mAb 3G8 (Biolegend, San Diego, CA) and the Pierce direct immunopreciptation kit (Thermo Fisher Scientific, Rockford, IL), as per the manufacturer’s instructions. Purified CD16 was deglycosylated by chitin binding domain-tagged Remove-iT PNGase F (New England BioLabs, Ipswich, MA), as per the manufacturer’s instructions. Briefly, 10–20μg of purified CD16 was denatured in the presence of 40mM DTT at 55°C for 10 minutes and then incubated with 3μl of Remove-iT PNGase F at 37°C for 1 hour. Remove-iT PNGase F was then removed from the reaction using chitin magnetic beads (New England BioLabs). CD16 was subjected to SDS-PAGE and gel bands corresponding to soluble CD16 were detected by a Krypton Fluorescent Protein Stain (Thermo Fisher Scientific), verified by CD16 immunoblot analysis of adjacent lanes in the same gel, and were then excised and subjected to standard in-gel digestion with trypsin. Digested peptides extracted from the gel were dried down and reconstituted for liquid chromatography-mass spectrometry analysis in 98:2:0.01, water:acetonitrile:formic acid and ≤1μg aliquots were analyzed on a Velos Orbitrap MS system (Thermo Fisher Scientific) in a data dependent scan mode, as described previously [18]. Database searches were performed with Protein Pilot 4.5 (AB Sciex, Foster City, CA), which uses the Paragon scoring algorithm [19], against the NCBI reference sequence Homo sapiens protein FASTA database to which the contaminant database (thegpm.org/cRAP/index,109 proteins) was appended. Search parameters were: cysteine iodoacetamide; trypsin; instrument Orbi MS (1–3ppm) Orbi MS/MS; biological modifications ID focus, which includes asparagine deamidation; a thorough search effort; and False Discovery Rate analysis (with reversed database).

### Generation of cDNA expression constructs

CD16b occurs as two allelic variants termed NA1 and NA2, differing by 4 amino acids in the N-terminal portion of its extracellular region [1, 20]. We have shown that both allelic variants of CD16b are cleaved with similar efficiency by ADAM17 [9]. For this study, we examined only the NA1 variant. There are also two allelic variants of CD16a that have either a valine or phenylalanine residue at position 176 [21]. We found that these two allelic variants of CD16a were also cleaved with similar efficiency by ADAM17 (data not shown). For this study, we examined only the valine allelic variant of CD16a. CD16a and CD16b were amplified from human leukocyte cDNA, separately cloned into the pcDNA3.1 plasmid (Invitrogen, Carlsbad, CA) at the *BamHI* and *EcoRI* restriction enzyme sites (restriction enzymes were purchased from New England BioLabs), as previously described [9, 22]. The constructs were then subjected to Quik-Change Site-directed Mutagenesis (Agilent Technologies, Santa Clara, CA) per the manufacture’s instructions to convert the serine at position 197 to a proline in CD16a and CD16b. All constructs were sequenced to confirm the presence of the intended mutation and the absence of any spontaneous mutations. The CD16a cDNA was subsequently cloned into the bi-cistronic retroviral expression vector pBMN-IRES-EGFP, provided by Dr. G. Nolan (Stanford University, Stanford, CA), at the *BamHI* and *EcoRI* restriction enzyme sites. The CD16a constructs were also cloned into a bicistronic *Sleeping Beauty* transposon plasmid (pKT2-IRES-GFP:zeo) that we have previously utilized [23, 24]. Briefly, wild-type CD16a and CD16a/S197P were PCR amplified using the primers: 5'-CCG GAA TTC CAG TGT GGC ATC ATG TGG CAG CTG CTC-3' (sense) and 5'-CCG GAA TTC TCA TTT GTC TTG AGG GTC CTT TCT-3' (antisense). *EcoRI* sites are underlined. The *EcoRI*-digested CD16a and CD16a/S197P PCR fragments were separately cloned into pKT2-IRES-GFP:zeo. Correct CD16a orientation and sequence were confirmed by PCR and sequencing analyses. We have previously cloned full-length human L-selectin (CD62L) cDNA [25, 26], which was transferred to the pcDNA3.1 vector at the restriction enzyme site *Xba1*. Full-length human FcRγ cDNA was cloned as previously described [22], with the modification that a pcDNA3.1 vector was used.

### Generation of cell lines expressing recombinant L-selectin, CD16a, and CD16b

HEK293 cells (a human embryonic kidney cell line) and NK92 cells (a human NK cell line) (ATCC, Manassas, VA) were cultured per the company’s instructions. HEK293 cells were transiently transfected with pcDNA3.1 with or without CD16b, CD16b/S197P, and/or L-selectin using Lipofectamine 2000 (Invitrogen) per the manufacturer’s instructions. HEK293 cells stably expressing human FcRγ were transiently transfected with pcDNA3.1 with or without CD16a or CD16a/S197P by the same approach. NK92 cells were stably transduced with pBMN-IRES-EGFP with or without CD16a or CD16a/S197P by retrovirus generation and infection procedures described previously [26–28]. Construct expression was assessed by EGFP fluorescence and CD16 staining, as determined by flow cytometry. Human iPSCs (UCBiPS7, derived from umbilical cord blood CD34 cells) were maintained on mouse embryonic fibroblasts [29, 30]. Stable expression of CD16a or CD16a/S197P was performed using a *Sleeping Beauty* transposon system, as previously described [23, 24]. Briefly, iPSCs were nucleofected with pKT2-IRES-GFP:zeo in combination with transposase DNA in nucleofector solution V (Lonza Inc., Gaithersburg, MD) using program setting B16. Nucleofected cells were immediately suspended in iPSC growth medium containing zeocin (50μg/ml) and seeded onto mouse embryonic fibroblasts.

### NK cell derivation from iPSC cells

A spin embryoid body (spin EB) approach was used for hematopoietic differentiation of iPSCs. At day 11 of hematopoietic differentiation, spin EBs were transferred into 24-well plates with EL08-1D2 stromal cells in NK media supplemented with cytokines, which produces phenotypically mature and functional NK cells, as previously demonstrated by our group [29, 30]. After 4 weeks of culture, single cell suspensions were stained for various phenotypic markers, including CD16, CD45, CD56, NKG2D-PE, NKp44, NKp46, CD158b, CD158e1/2 (BD Biosciences, San Jose, CA), CD158a/h and CD158i (Beckman Coulter, Indianapolis IN), as previously described [29, 30]. Antibody staining was assessed by flow cytometry.

### Cell stimulation

HEK293 and NK92 cells in RPMI 1640 media (Mediatech) were activated with 15ng/ml and 100ng/ml, respectively, PMA for 30 minutes at 37°C. NK92 cells were activated with IL-12 (PeproTech Inc, Rocky Hill, NJ) and IL-18 (R&D Systems, Minneapolis, MN) at 100 ng/ml and 400 ng/ml, respectively, for the indicated time points. NK92 cell activation through CD16a was mediated by their incubation with the CD20-positive Burkitt’s lymphoma cell line Raji (ATCC, grown per the company’s instructions) (1:1 ratio) treated with the anti-CD20 mAb rituximab (1μg/ml) (Genentech, South San Francisco, CA), as described previously [10]. Excess rituximab was removed by washing the Raji cells. In some experiments, NK92 cells were pre-incubated for 30 minutes with the selective ADAM17 inhibitor BMS566394 (5μM) (Bristol-Myers Squibb Company, Princeton, NJ), which is referred to as inhibitor 32 in reference [31]. NK cells derived from iPSCs were stimulated with the human erythroleukemic cell line K562 (ATCC, grown per the company’s instructions), as previously described [10]. Briefly, iPSC-derived NK cells were incubated with K562 target cells (2:1 ratio) for 4 hours at 37°C.

### Antibody binding assay

Cell binding to monomeric human IgG and IgA (Sigma) was performed as previously described with some modifications [22]. NK92 parent cells or transduced cells expressing CD16a or CD16a/S197P at 5x10^6^/ml in PBS were incubated with IgG or IgA at the indicated concentrations in triplicate for 1 hour at 4°C. The cells were extensively washed and incubated with APC-conjugated donkey anti-human Fc (heavy and light chain) antibody (Jackson Immunoresearch, West Grove, PA), as per the manufacture’s instructions. The cells were washed and then immediately analyzed by flow cytometry.

### Flow cytometry and ELISA

For cell staining, nonspecific antibody binding sites were blocked and cells were stained with the indicated antibodies and examined by flow cytometry, as previously described [9, 10]. Flow cytometric analysis was performed on FACSCanto and LS RII instruments (BD Biosciences). Human CD16 was detected by the mAbs 3G8 (Biolegend) and DJ130c (Santa Cruz Biotech, Santa Cruz, CA). CD107a was detected by the mAb H4A3 (Biolegend). ADAM17 was detected by the mAbs M220 [32], 111633, and 111623 (R&D Systems). Human L-selectin was detected by the mAb LAM1-116 (Ancell, Bayport, MN). Isotype-matched negative control mAbs were used to evaluate levels of nonspecific staining. The CD16 ELISA was performed by a custom cytometric bead assay, as previously described [9].

### Statistical analysis

Statistical analysis was performed using Prism software (GraphPad, San Diego, CA) using ANOVA and student’s t test where appropriate. A p value of < 0.05 was considered significant.

## Results

### Identification of CD16 cleavage sites

**Fig 1:**
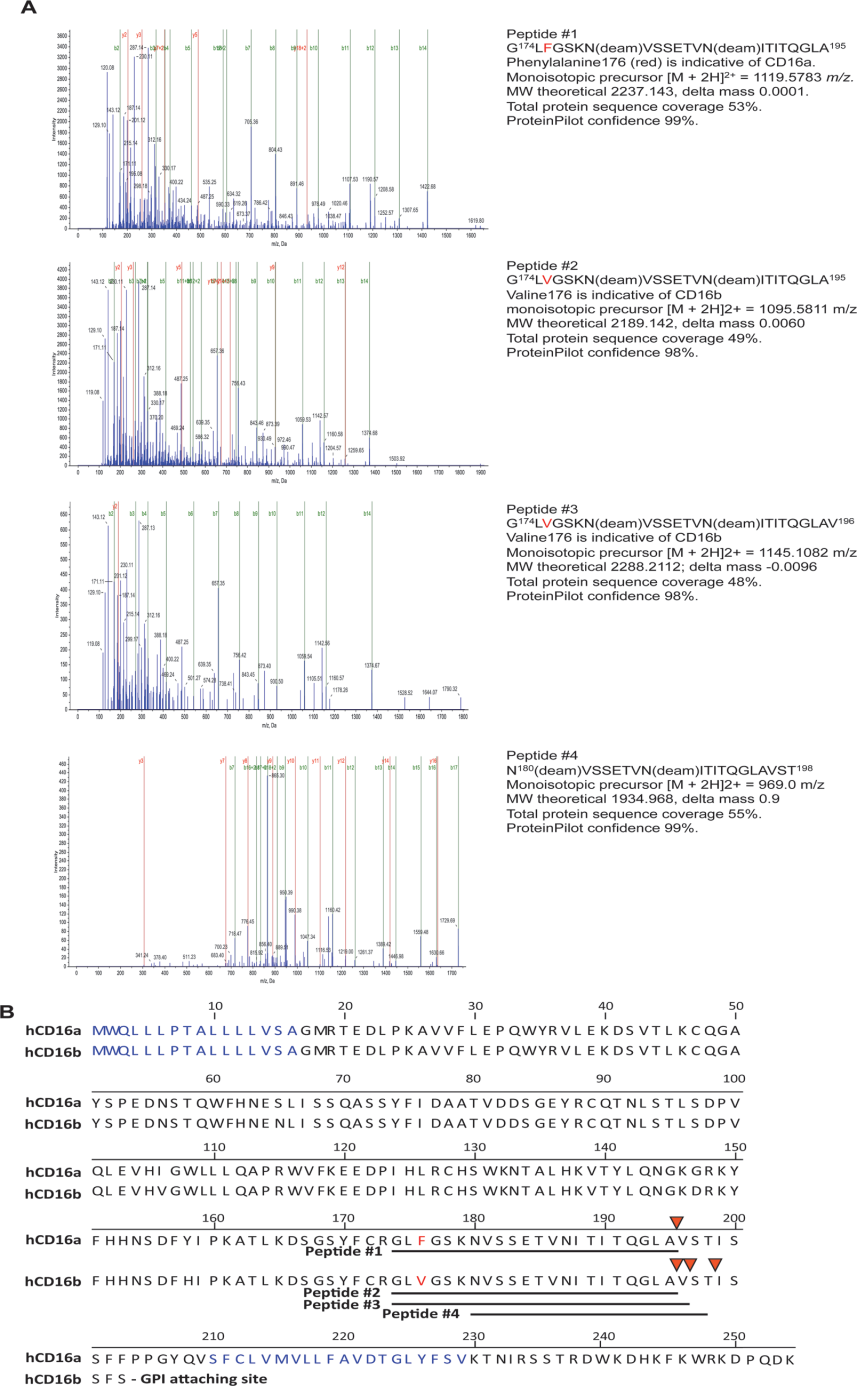
Location of ectodomain cleavage sites in human CD16. (A) Tryptic peptides of soluble CD16 immunoprecipitated from the cell supernatant of PMA activated human NK cells or neutrophils were subjected to mass spectrometry analysis. Four high confidence peptides with non-tryptic C-termini were identified; 1 peptide from soluble CD16 released by NK cells (Peptide #1) and 3 peptides from soluble CD16 released by neutrophils (Peptides #2–4). (B) Illustration of Peptides #1–4 (underlined) and putative cleavage sites (arrowheads) in CD16a and CD16b. The red amino acids indicate residues that distinguish CD16a and CD16b in the identified peptides. Blue amino acids indicate predicted signal sequences of CD16a and CD16b and the transmembrane region of CD16a. Amino acid numbering begins with methionine in the signal sequence. The amino acid sequences of CD16a and CD16b are from the NCBI reference sequences NM_000569.6 and NM_000570.4, respectively.

**Fig 2:**
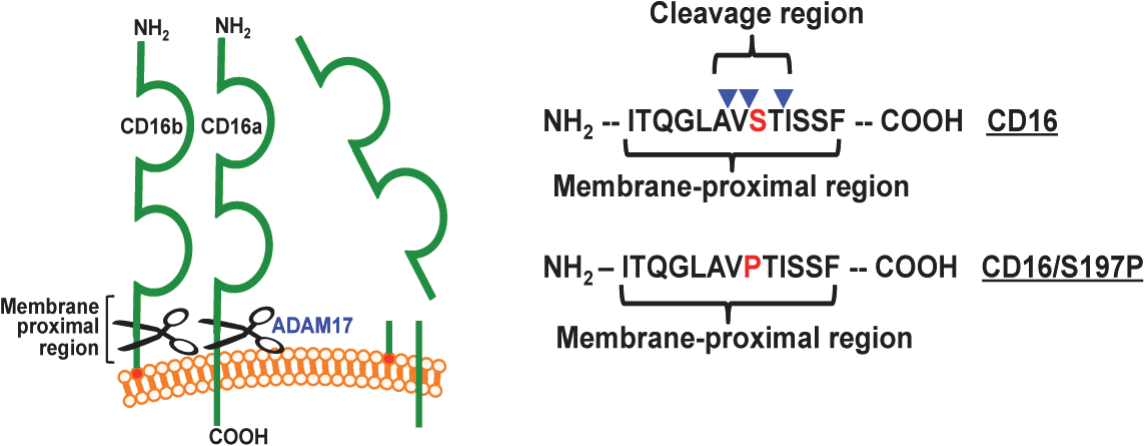
Schematic illustration of CD16 ectodomain shedding, the cleavage region, and the engineered serine197 to proline mutation. CD16a and CD16b undergo ectodomain shedding by ADAM17 within a membrane proximal region, as indicated. The CD16 cleavage region within the membrane proximal region is based on mass spectrometry analysis that revealed three distinct cleavage sites in close proximity (arrowheads). Site-directed mutagenesis was performed to exchange serine at position 197 for a proline (S197P), as indicated in red font.

ADAM17 has been reported to cleave CD16a and CD16b from the surface of human NK cells and neutrophils, respectively [9, 10]. We investigated the location of CD16 cleavage by separately immunoprecipitating this protein from the media supernatant of activated NK cells or neutrophils. Immunoprecipitated CD16 was then treated with PNGaseF to remove N-glycans, trypsin digested, and the generated peptides subjected to mass spectrometric analysis. Four different peptide patterns of high confidence were identified containing non-tryptic C-termini (Fig 1A). For CD16 enriched from the media supernatant of activated NK cells we observed only one peptide pattern, which consisted of the amino acids glycine174—alanine195 (Peptide #1, Fig 1A). The membrane proximal regions of CD16a and CD16b have identical amino acid sequences except for residue 176. A phenylalanine at this location is indicative of CD16a, which was present in Peptide #1 (Fig 1A and 1B). This peptide revealed a non-tryptic P1/P1′ cleavage position at alanine195/valine196 (Fig 1B). For CD16 enriched from the media supernatant of activated neutrophils, we detected three different peptide patterns with non-tryptic C-termini (Peptides #2–4, Fig 1A). Two of the peptides contained a valine at position 176, indicative of CD16b, and revealed P1/P1′ positions at alanine195/valine196 and at valine196/serine197 (Fig 1B). The forth peptide spanned from asparagine180 to threonine198, thus revealing a P1/P1′ position at threonine198/isoleucine199 (Fig 1B). Though this peptide was derived from soluble CD16 from enriched neutrophils, it does not contain an amino acid at position 176 to identify the isoform (Fig 1B). Regardless, the high confidence peptide revealed a third cleavage site in CD16. Taken together, these findings demonstrate the presence of a cleavage region in CD16, and not a single specific cleavage site, as depicted in Fig 2.

### Site-directed mutagenesis within the CD16 cleavage region

We further examined the cleavage region in CD16 by using site-directed mutagenesis to determine whether CD16a and CD16b cleavage could be disrupted in cell-based assays, which has not been previously reported. ADAM17 tends to prefer an α-helical conformation in the substrate region that interacts with its catalytic site [33, 34]. Moreover, proteomics studies of ADAM17 cleavage site specificities revealed a very low preference for proline residues at the P1′, P2′ or P3′ positions [35–37]. In accordance with this, we substituted serine-197 in the cleavage regions of CD16a and CD16b with a proline (S197P, as indicated in Fig 2).

**Fig 3:**
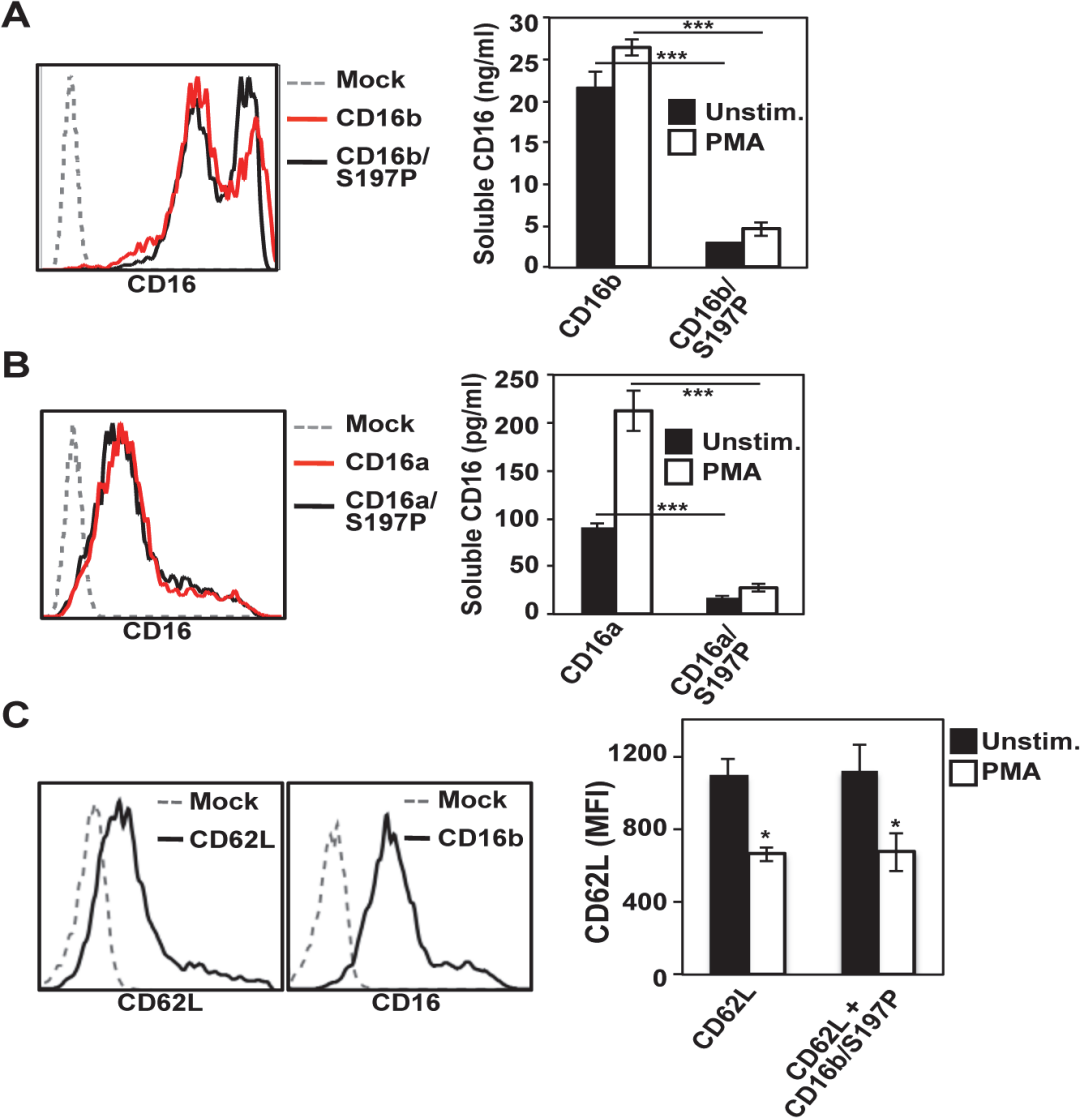
Effects of the engineered S197P mutation on CD16a and CD16b shedding. Transfected HEK293 (human embryonic kidney) cells separately expressed CD16b and CD16b/S197P (A) or CD16a and CD16a/S197P (B) at similar levels, as determined by flow cytometry (left panels). The different transfectants were treated with or without PMA (15ng/ml for 30 minutes at 37°C) and soluble levels of CD16 in the media supernatant were quantified by ELISA (right panels). Each treatment condition was repeated 3 times for each experiment and the data are representative of 3 independent experiments. Bar graphs show mean ± SD. Statistical significance is indicated as ***P<0.001. (C) Transfected HEK293 cells expressed L-selectin (CD62L) or L-selectin and CD16b/S197P. Surface levels of L-selectin and CD16b/S197P on transfected and mock-transfected cells were measured using flow cytometry (histogram plots). Transfectants expressing L-selectin or L-selectin and CD16b/S197P were incubated in the presence or absence of PMA for 30 minutes at 37°C, and the mean fluorescence intensity (MFI) of L-selectin staining determined (bar graph). Each treatment condition was repeated 3 times for each experiment and the data are representative of 2 independent experiments. Bar graphs show mean ± SD. Statistical significance is indicated as *P<0.05. For all histogram plots, the x-axis = Log 10 fluorescence and the y-axis = cell number.

CD16b and CD16b/S197P were separately expressed in the human kidney cell line HEK293, which does not express endogenous CD16. The HEK293 transfectants expressed CD16b or CD16b/S197P at similar levels on their surface (Fig 3A). High levels of CD16b were released from the transfected HEK293, presumably due to their transformed state [38], which was increased further upon their treatment with PMA, as determined by ELISA (Fig 3A). However, soluble levels of CD16b/S197P generated by untreated or PMA-treated HEK293 cells were markedly lower than those of CD16b (Fig 3A). We also examined the effects of the S197P mutation on CD16a cleavage using the same approach. Surface expression of CD16a requires association with γ chain dimers [4], and therefore we used HEK293 cells stably expressing human γ chain. Comparing HEK293 transfectants expressing equivalent surface levels of CD16a or CD16a/S197P (Fig 3B), we determined the soluble levels of each receptor in the media supernatant of untreated and PMA-treated cells. Again, significantly lower levels of soluble CD16a/S197P were observed when compared to CD16a (Fig 3B). To evaluate whether the engineered S197P mutation in CD16 might disrupt ADAM17’s activity, we also transfected HEK293 cells expressing or lacking CD16b/S197P with L-selectin, a well described ADAM17 substrate normally expressed by leukocytes [39, 40]. Both transfectants expressed equivalent levels of L-selectin, which was similarly down-regulated following their activation with PMA (Fig 3C), demonstrating that the S197P mutation affected CD16 shedding and not ADAM17 activity.

**Fig 4:**
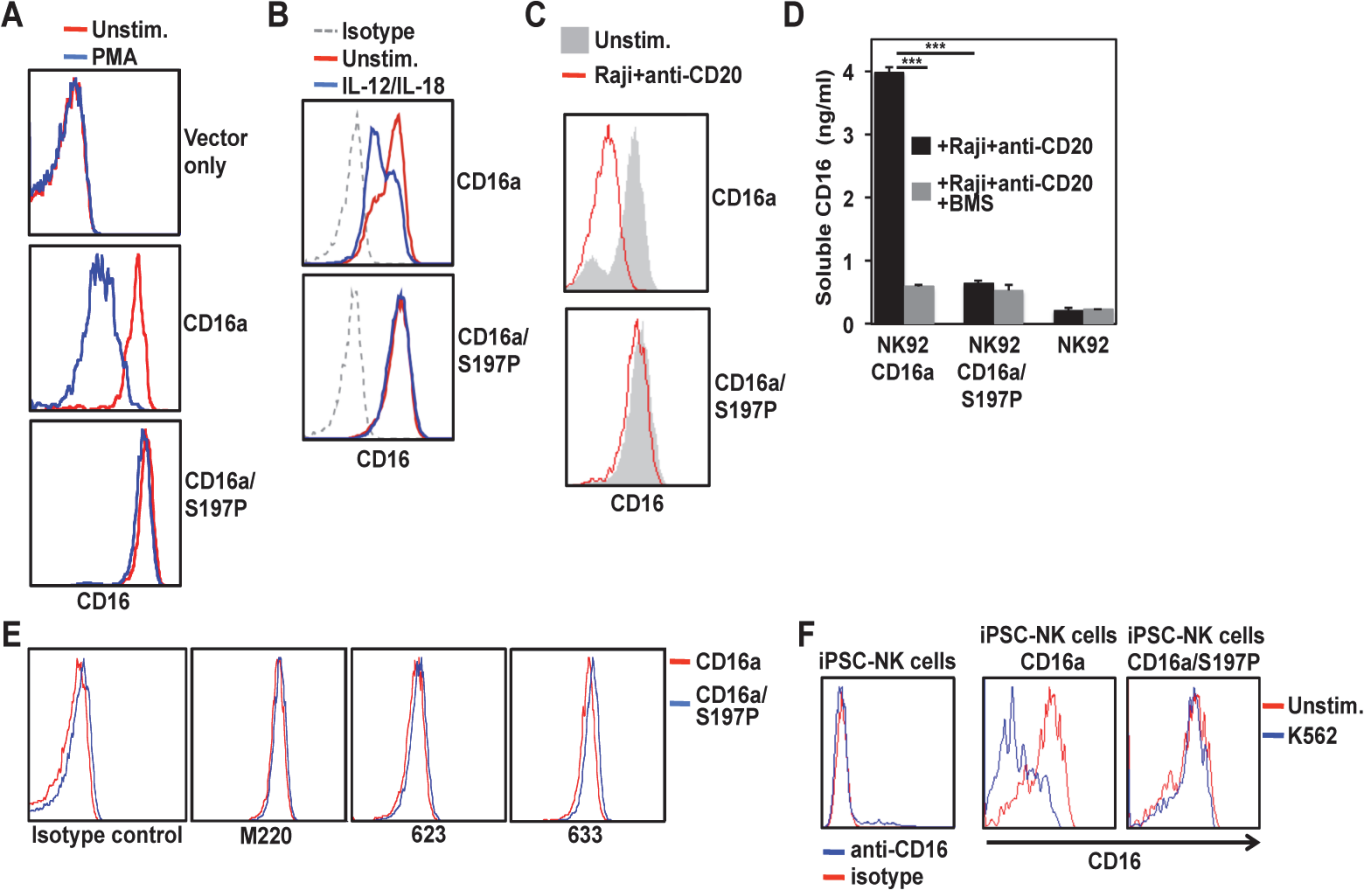
Effects of the engineered S197P mutation on CD16a shedding in NK cells. NK92 cells transduced with empty vector (vector only), CD16a, or CD16a/S197P were treated without (Unstim.) or with PMA (100ng/ml) for 30 minutes at 37°C (A), with IL-12 and IL-18 (100ng/ml and 400ng/ml, respectively) for 24 hours at 37°C (B), or with Raji cells and rituximab for 60 min at 37°C (C). Cell surface levels of CD16a were determined by flow cytometry. Isotype-matched negative control antibody staining is indicated by a dotted line. (D) Parent NK92 cells and transduced cells expressing CD16a or CD16a/S197P were treated with Raji cells and rituximab in the presence or absence of the ADAM17 inhibitor BMS566394 (5μM) for 60 min at 37°C. Soluble CD16a levels were determined by ELISA. Each treatment condition was repeated 3 times and the data are representative of 3 independent experiments. Bar graphs show mean ± SD. Statistical significance is indicated as ***P<0.001. (E) NK92 cells expressing CD16a or CD16a/S197P were stained with the anti-ADAM17 mAbs M220, 623, 633, or an isotype-matched negative control antibody, as indicated. (F) CD56^+^CD45^+^ NK cells derived from mock-transduced iPSCs (left panel) or iPSCs expressing recombinant CD16a or CD16a/S197P (right panels) were incubated with or without K562 target cells for 4 hours at 37°C. For all histogram plots, the x-axis = Log 10 fluorescence, the y-axis = cell number, and the data are representative of at least 3 independent experiments.

To assess the effects of the S197P mutation on CD16a shedding in NK cells, we used the human NK cell line NK92 [41]. These cells lack expression of endogenous CD16a, but recombinant CD16a can be stably expressed [42]. We transduced NK92 cells to separately express CD16a and CD16a/S197P. Cells expressing equivalent levels of these receptors were activated with PMA and cell surface CD16 levels were examined by flow cytometry. We found that CD16a, but not CD16a/S197P, underwent a marked down-regulation in expression (Fig 4A). IL-12 and IL-18 are important physiological stimuli of NK cells that individually or in combination induce CD16a shedding [10]. NK92 cells treated with IL-12 and IL-18 demonstrated an appreciable down-regulation in their expression of CD16a but not CD16a/S197P (Fig 4B). Direct engagement of cell bound IgG by CD16a also induces its shedding [10], which we examined here by incubating NK92 cells expressing CD16a or CD16a/S197P with the CD20-positive Burkitt’s lymphoma cell line Raji in the presence or absence of the anti-CD20 mAb rituximab. Consistent with other stimuli, Raji cells treated with rituximab induced the down-regulation of CD16a, but not CD16a/S197P (Fig 4C).

BMS566394 is a highly selective ADAM17 inhibitor with a potency orders of magnitude higher for ADAM17 than for other metalloproteases [9, 31, 43]. We show that BMS566394 blocked CD16a shedding with similar efficiency as the S197P mutation, but had no additional blocking effect on activated NK92 cells expressing CD16a/S197P (Fig 4D). These findings provide further evidence that ADAM17 is the primary sheddase that cleaves CD16a within its cleavage region. It is possible, however, that ADAM17 expression levels were not equivalent in the NK92 cells expressing CD16a or CD16a/S197P, accounting for their dissimilar shedding. We therefore stained NK92 cells expressing CD16a or CD16a/S197P with multiple anti-ADAM17 mAbs and observed identical cell surface levels (Fig 4E).

To establish the effect of the S197P mutation on CD16a shedding by primary NK cells, we used human iPSCs to generate engineered NK cells. We have previously reported on deriving functional NK cells from iPSCs and their similarity to peripheral blood NK cells [29, 30]. CD16a and CD16a/S197P cDNA were cloned into a *Sleeping Beauty* transposon plasmid for gene insertion and stable expression in iPSC cells, which were subsequently differentiated into mature NK cells. NK cells derived from mock transduced iPSC cells expressed low levels of endogenous CD16a, whereas transduced CD16a and CD16a/S197P were expressed at higher levels (Fig 4F). NK cell activation occurs through various receptors upon their interaction with K562 cells, including BY55/CD160 [44], resulting in ADAM17 activation and CD16a shedding [10]. We stimulated the iPSC-derived NK cells with K562 cells and found that CD16a underwent a marked down-regulation in expression, whereas the expression of CD16a/S197P remained stable (Fig 4F).

### Effects of the S197P mutation on CD16a function

**Fig 5:**
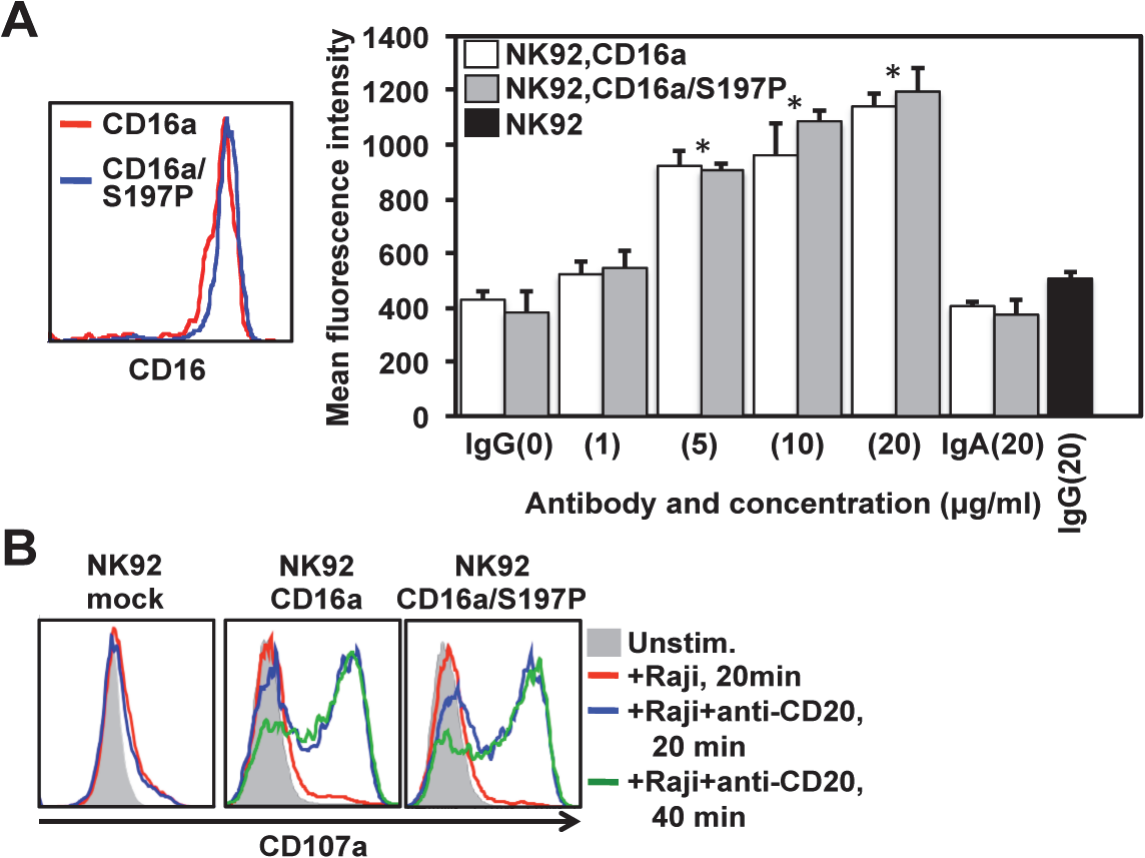
Effects of the engineered S197P mutation on CD16a function. (A) NK92 cells expressing CD16a or CD16a/S197P at equivalent levels (left panel) were treated with monomeric human IgG (0–20μg/ml). As controls, cells were also treated with monomeric human IgA (20μg/ml), and NK92 parent cells were treated with IgG (20μg/ml) (black bar). Antibody binding was determined by flow cytometry, as described in Materials and Methods. The bar graph shows mean ± SD of at least 3 separate experiments. Statistical significance is indicated as *P<0.05 versus IgG (0 μg/ml), IgA, or NK92 parent cells + IgG. (B) Mock transduced NK92 cells or NK92 cells expressing CD16a or CD16a/S197P were incubated in the absence (Unstim.) or presence of Raji cells treated with or without anti-CD20 rituximab for the indicated time points at 37°C. NK92 cell activation was assessed by the up-regulation in CD107a staining by flow cytometry. For the histogram plots, the x-axis = Log 10 fluorescence and the y-axis = cell number. Data are representative of at least 3 independent experiments.

Endogenous and recombinant CD16a are known to have sufficient affinity to bind monomeric IgG [21, 22]. To examine the effects of the S197P mutation on CD16a function, we compared the IgG binding capacities of CD16a and CD16a/S197P. We found that NK92 cells expressing CD16a or CD16a/S197P at equivalent levels bound IgG in a similar dose-dependent manner (Fig 5A). Controls consisted of IgA binding to NK92 cells expressing CD16a or CD16a/S197P, and IgG binding to NK92 parent cells. Both occurred at essentially background levels (Fig 5A). These findings demonstrate specific and equivalent IgG binding by CD16a and CD16a/S197P.

CD16a is a potent activating receptor in NK cells [45, 46], and we examined whether the engineered S197P mutation affected the capacity of CD16a to induce cell activation upon engagement of antibody-treated tumor cells. NK92 cell activation was assessed by measuring the up-regulation of CD107a, which occurs very rapidly upon degranulation and is a sensitive marker of NK cell activation [47]. Mock transduced NK92 cells incubated with Raji cells treated with or without rituximab demonstrated low level and similar up-regulation CD107a (Fig 5B), which is not unexpected considering that NK92 cells express various activating receptors [41]. NK92 cells expressing CD16a or CD16a/S197P at equivalent levels when incubated with Raji cells alone marginally up-regulated CD107a as well, whereas their incubation with Raji cells treated with rituximab resulted in a considerable up-regulation of CD107a (Fig 5B). Taken together, the above findings indicate that the engineered S197P mutation in CD16a did not impair its function.

## Discussion

CD16a and CD16b undergo very rapid and efficient ectodomain shedding from the surface of human leukocytes following their activation by diverse stimuli [6–10]. Our findings demonstrate for the first time a cleavage region in CD16 (Fig 2). We identified a single cleavage site in CD16a released from NK cells at P1/P1′ position alanine195/valine196. This finding is in agreement with peptide library-based analyses of ADAM17 cleavage sites that demonstrated the protease’s preference for a valine residue at the P1′ position [35, 36]. Recombinant ADAM17 was also found to cleave a synthesized peptide corresponding in sequence with the membrane proximal region of CD16 (both CD16a and CD16b) at the P1/P1′ position alanine195/valine196 [12]. Additionally, we identified 3 cleavage sites in CD16b from neutrophils at the P1/P1′ positions alanine195/valine196, valine196/serine197, and threonine198/isoleucine199. Of interest is that the valine196/serine197 cleavage position was previously identified upon examination of soluble CD16 in human plasma [48]. A serine at the P1′ position also occurs in the well-characterized ADAM17 substrate L-selectin [49, 50]. Our findings suggest that there may be greater variability at where ADAM17 cleaves CD16b than CD16a. An important difference between the two receptors is that CD16b is GPI linked to the plasma membrane [2], and perhaps this causes more fluctuation in the interaction of its membrane proximal region with the catalytic domain of ADAM17.

To further establish the significance of the CD16 cleavage region, serine197 in the middle of this region was exchanged for a proline residue. Proteomics studies of ADAM17 cleavage site specificities indicate a very low preference for a proline residue at the P1′, P2′ or P3′ positions, though this appears not to be the case for the P1 position [35–37]. CD16 shedding does not occur in mouse leukocytes and, incidentally, the amino acid corresponding to serine197 in human CD16 is a proline in mouse CD16 [9]. We show that the engineered S197P mutation in CD16a and CD16b effectively blocked their shedding in cell-based assays that involved native ADAM17.

The S197P mutation in CD16a also blocked shedding of the receptor in the human NK cell line NK92, but it did not impair receptor function. The latter was determined by comparing the IgG binding and cell activation capacities of wild-type CD16a and CD16a/S197P. We show that NK92 cells expressing equivalent levels of CD16a or CD16a/S197P bound monomeric IgG with similar efficiency over a range of antibody concentrations. In addition, NK92 cells expressing CD16a or CD16a/S197P up-regulated the activation marker CD107a in a comparable manner upon their engagement of rituximab bound to Raji cells.

Pluripotent stem cells offer the critical advantage of genetic manipulation to generate engineered NK cells [51]. We and others have produced iPSC cell lines from a variety of somatic cell populations and have demonstrated efficient hematopoietic development from these cells [52–54], including the generation of mature NK cells that express activating and inhibitory receptors similar to peripheral blood NK cells and that kill tumor targets [29, 30]. We report here the generation of engineered NK cells from transduced iPSCs expressing wild-type CD16a or CD16a/S197P. As with NK92 cells, CD16a underwent shedding in the iPSCs-derived NK cells, demonstrating normal ADAM17 activity upon cell activation, whereas CD16a/S197P was not shed.

It has been reported that CD16a and NK cell cytotoxic function can undergo a considerable down-regulation in cancer patients [55, 56]. It will be important to determine in future studies whether the engineering of NK92 cells or primary NK cells derived from pluripotent stem cells to express CD16a/S197P will prevent the down-regulation of this key FcγR upon cell activation in the tumor environment and enhance their ADCC potency and cancer cell killing.
